# Constipation and Allied Intestinal Disorders

**Published:** 1910-06

**Authors:** 


**Constipation and allied Intestinal Disorders.** By Arthur F. Hertz, M.D. Oxon. Pp. xiii, 344. London : Oxford Medical Publications. 1909.

Three years' research on the physiology and pathology of the movements of the alimentary canal, with the assistance of grants from the British Medical Association and the help of many friends, particularly in the X-ray investigations, have enabled the author to produce a work which should be an advance on the previous monographs on constipation. Many attempts to determine what part of the intestine is to blame in different forms of constipation have been made by tracing with the X-rays the passage of food mixed with a bismuth salt, and by comparing the results so obtained with the clinical symptoms and signs much useful knowledge has been gained. Bismuth has been given in much larger doses than heretofore, two ounces of the bismuth oxy-chloride having taken the place of much smaller doses of the bismuth subnitrate, which in full doses has not seldom given rise to symptoms of poisoning. The oxychloride is quite inert in the stomach, and is also uninfluenced by the alkaline intestinal contents. After a bismuth breakfast at 8 a.m., the shadow reaches the splenic flexure by five in the afternoon and the pelvic colon the next morning.

The causes of constipation are classified according to an elaborate system into (*a*) deficient motor activity of the intestines, (*b*) obstruction of the intestinal lumen, (*c*) inefficient defæcation, and (*d*) obstructed defæcation. The last two classes are grouped together under the term Dyschezia. In the majority of the cases of primary constipation, Dr. Hertz has found that the stasis occurs in the pelvic colon, and he considers that the whole colon below the splenic flexure should be emptied at each act of defæcation. The idea that the cæcum and ascending colon ever form a " cesspool " is strongly denied. These facts cause the author to condemn the treatment of severe constipation by operative methods. But he overlooks the fact that the operation of ileo-sigmoidostomy is one which results in fluid being passed into the pelvic colon instead of hard fæces, and it may well be that this gut can deal with such contents effectively when it is powerless to expel scybala. The fact that in idiopathic dilatation of the colon a larger proportion of cases treated surgically recover than of those treated by expectant methods is not mentioned.

Rather more than half the book is occupied by a consideration of the symptoms and treatment of constipation, and whilst this forms a most able and valuable résumé of what is known on this subject, one is rather struck with the paucity of new ideas of practical importance which have resulted from the new method of investigation.

